# Efficacy, Effectiveness, and Safety of Treatment Regimens for Histoplasmosis and Tuberculosis Coinfection in Patients With Human Immunodeficiency Virus/Acquired Immunodeficiency Syndrome: A Systematic Review With Meta‐Analysis Protocol

**DOI:** 10.1002/hsr2.71720

**Published:** 2026-01-06

**Authors:** Beatriz Bernava Sarinho, Inajara Rotta, Tácio de Mendonça Lima, Brígida Dias Fernandes, Patricia Melo Aguiar, Marília Berlofa Visacri

**Affiliations:** ^1^ Department of Pharmacy, School of Pharmaceutical Sciences University of São Paulo São Paulo SP Brazil; ^2^ Department of Pharmacy Federal University of Paraná Curitiba PR Brazil; ^3^ Department of Pharmacy and Pharmaceutical Administration, Faculty of Pharmacy Fluminense Federal University Niteroi RJ Brazil; ^4^ Capixaba Institute of Education Research and Innovation in Health Vitória ES Brazil

**Keywords:** AIDS‐related opportunistic infections, histoplasmosis, meta‐analysis, systematic review, treatment outcome, tuberculosis

## Abstract

**Background and Aims:**

While previous publications have briefly pointed out a few alternative therapeutic interventions for the treatment of histoplasmosis and tuberculosis coinfections in patients with Human Immunodeficiency Virus (HIV) and Acquired Immunodeficiency Syndrome (AIDS), there are no reviews evaluating the efficacy, effectiveness, and safety of different regimens. Thus, this protocol outlines the approach for systematically reviewing and synthesizing the existing literature regarding the efficacy, effectiveness, and safety of different regimens for histoplasmosis and tuberculosis coinfection treatment in patients with HIV/AIDS.

**Methods:**

A systematic search will be conducted using PubMed, Embase, and Latin American and Caribbean Health Sciences Literature (LILACS) databases. Studies will be selected in two distinct stages, and data from selected studies will be extracted. Risk of bias will be assessed using the RoB 2, ROBINS‐I, NOS, and JBI tools, depending on the type of study. Finally, the strength of the body of evidence will be assessed using the Grading of Recommendations Assessment, Development and Evaluation (GRADE) methodology.

**Conclusions:**

The findings of this review may positively impact public health, stimulating the implementation of evidence‐based practices and guiding the adoption of specific treatment protocols for patients with HIV/AIDS coinfected with histoplasmosis and tuberculosis in endemic regions.

## 1 Introduction

Immunocompromised patients, such as people living with the Human Immunodeficiency Virus (HIV) and Acquired Immunodeficiency Syndrome (AIDS), commonly present opportunistic infections, especially tuberculosis (TB) and histoplasmosis [1, 2]. In a Brazilian study, TB infection was identified as the main cause of death (28%) among people living with HIV/AIDS, with deaths resulting from histoplasmosis infections also being prevalent (13%) [3]. In endemic regions to both opportunistic infections, co‐occurrence is common, and it is estimated that 8%–15% of HIV‐infected patients are coinfected with histoplasmosis and TB [4]. In 2024, a prospective Paraguayan study identified histoplasmosis‐TB coinfection as the most common among people living with HIV/AIDS, associated with 70% of coinfection cases. As a result of non‐specific symptoms, overlapping radiological abnormalities, and high associated diagnostic costs, histoplasmosis identification often does not occur or is delayed, typically happening when symptoms persist after TB treatment [2, 4].

The first‐line antiretroviral therapy (ART) regimen for HIV management consists of a combination of three drugs—tenofovir, lamivudine and dolutegravir. For an alternate first‐line ART, dolutegravir may be replaced by efavirenz [5]. In parallel, first‐line treatment for drug‐susceptible TB consists of a 6‐month regimen called 2HRZE/4HR, or RIPE, in which 2 months of daily isoniazid, rifampicin, ethambutol, and pyrazinamide are followed by 4 months of daily isoniazid and rifampicin. As an alternative, a 4‐month regimen (2PHZM/2PHM) may be used, where 8 weeks of daily isoniazid, rifapentine, moxifloxacin and pyrazinamide are followed by 9 weeks of daily isoniazid, rifapentine, and moxifloxacin [6]. Lastly, for the treatment of mild to moderate histoplasmosis cases in people living with HIV/AIDS, induction therapy with itraconazole is recommended, while liposomal amphotericin B is recommended for induction in severe or moderately severe histoplasmosis. Maintenance therapy with itraconazole is recommended regardless of severity [7].

However, treatment of histoplasmosis‐TB coinfections is challenging, as drug‐drug interactions occur between itraconazole and rifampicin [2, 4]. Such interactions occur because rifampicin is a potent inducer of cytochrome P450 enzymes involved in itraconazole metabolism. Therefore, concomitant administration of both drugs is associated with the development of subtherapeutic blood concentrations of itraconazole, leading to ineffectiveness in histoplasmosis management. Additionally, interactions may also occur between the aforementioned drugs and antiretrovirals commonly used in HIV/AIDS management, notably those belonging to protease inhibitors and non‐nucleoside reverse transcriptase inhibitors classes [4, 8]. Table 1 shows the general effect of concomitant use on blood concentration for the drugs discussed.

**Table 1:** General effect of concomitant use on the blood concentration of the drugs discussed.

Combination*	General effect of concomitant use on blood concentration*
RIF/ITRA	↓ ITRA
RIF/PI	↓ PI
RIF/NNRTI	↓ NNRTI
ITRA/PI	↑ ITRA/ ↑ PI
ITRA/NNRTI	↑ NNRTI/ ↓ ITRA

Abbreviations: ITRA = itraconazole, NNRTI = non‐nucleoside reverse transcriptase inhibitors, PI = protease inhibitors, RIF = rifampicin,

* Department of Health and Human Services (HHS).

Given the drug–drug interactions that may occur, some therapeutic alternatives have been highlighted, such as replacing rifampicin with fluoroquinolones (e.g., moxifloxacin) and/or replacing itraconazole with amphotericin B or with newer antifungals that present fewer drug interactions (e.g., pozaconazole) [4]. Even so, while previous publications have briefly pointed out such alternative therapeutic interventions for histoplasmosis‐TB coinfection treatment in patients with HIV/AIDS, there are no reviews evaluating the efficacy, effectiveness, and safety of different regimens [4, 7]. Thus, aiming to address an important gap in the scientific literature and contribute to the improvement of clinical practice for the drug treatment of endemic and neglected clinical conditions, the primary objective of this systematic review is to evaluate the efficacy, effectiveness, and safety of different regimens for treating patients with HIV/AIDS coinfected with histoplasmosis‐TB.

This review aims to analyze the evidence available in the literature regarding:

a. The efficacy or effectiveness of different regimens for histoplasmosis‐TB coinfection treatment in patients with HIV/AIDS, based on clinical improvement, mortality, and relapse rates.
b. The safety of different regimens for histoplasmosis‐TB coinfection treatment in patients with HIV/AIDS, based on the frequency of adverse drug reactions and the proportion of patients who discontinue treatment due to these reactions.

## 2 Methods and Materials

### 2.1 Design

This systematic review will be reported according to the PRISMA (Preferred Reporting Items for Systematic Reviews and Meta‐Analyses Statement) 2020 statement [9]. The primary research question adopted for this review is: “What is the efficacy, effectiveness, and safety of different regimens for treating histoplasmosis‐TB coinfection in patients with HIV/AIDS?”

- P (Population)—patients with HIV/AIDS and histoplasmosis– TB coinfection.
- I (Intervention)—different treatment regimens for histoplasmosis–TB coinfection.
- C (Comparator)—different treatment regimens for histoplasmosis–TB coinfection, placebo, or lack of comparator.
- (Outcomes)—efficacy or effectiveness, safety.

The review protocol will also be registered to PROSPERO (https://www.crd.york.ac.uk/prospero/), and any deviations will be justified in the final report.

### 2.2 Eligibility Criteria

Interventional studies (randomized/non‐randomized controlled clinical trials, pre‐post); analytical observational studies (cohort, case‐control, cross‐sectional); and descriptive observational studies (case report, case series) that evaluate the efficacy, effectiveness, and safety of different regimens for histoplasmosis‐TB coinfection treatment in patients with HIV/AIDS and are published as journal articles will be considered eligible, without publication date restrictions. This inclusive approach is necessary given the relative scarcity of randomized trials including patients with HIV/AIDS and histoplasmosis‐TB coinfection.

Studies will be included if the diagnosis of TB and histoplasmosis was established using at least one of the diagnostic methods [10, 11] listed in Table 2, as these are recognized as reliable and validated tests for the detection of both infections. Only studies that report at least one of the outcomes described in Table 3 will be included.

**Table 2:** Validated and reliable diagnostic methods for tuberculosis and histoplasmosis included in study selection criteria.

Type of Test	Tuberculosis	Histoplasmosis
Microscopy	Ziehl–Neelsen staining (AFB smear); fluorescence microscopy with auramine‐rhodamine stain*	Direct examination with Wright–Giemsa, periodic acid–Schiff or Grocott–Gomori's methenamine silver stains**
Culture	Mycobacterial culture: Löwenstein–Jensen medium; BACTEC radiometric method; Septi–Chek AFB system; microcolony detection; MGIT*	Fungal culture on Sabouraud agar or brain–heart infusion agar supplemented with blood media**
Molecular tests	PCR or LAMP‐based assays for detection of *M. tuberculosis*; Xpert MTB/RIF; Xpert MTB/RIF Ultra*	PCR‐based assay for detection of *H. capsulatum*; AccuProbe test**
Antigen detection	LF‐LAM*	Histoplasma antigen detection in urine, serum, BAL or CSF samples (e.g., EIA tests)**
Antibody detection	Not applicable	Complement fixation; double immunodiffusion; ELISA; immunoblotting**

Abbreviations: AFB = acid‐fast bacilli, BAL = bronchoalveolar lavage, CSF = cerebrospinal fluid, EIA = enzyme immunoassay, ELISA = enzyme‐linked immunosorbent assay, LAMP = loop‑mediated isothermal amplification, LF‐LAM = lateral flow antigen lipoarabinomannan assay, MGIT = mycobacterium growth indicator tubes, PCR = polymerase chain reaction.

* Acharya et al. [10].

** Toscanini, Nusblat, Cuestas [11].

**Table 3:** Outcomes for studies on histoplasmosis‐tuberculosis coinfection treatment in patients with HIV/AIDS.

Outcome category	Specific outcomes		Definition/measurement
Efficacy/effectiveness	Clinical improvement		Improvement in signs/symptoms observed or documented in clinical records or scales
	Mortality	Early death	Death within 30 days from baseline evaluation
		Late death	Death after 30 days from baseline evaluation
	Relapse		Resurgence, after initial resolution, of signs/symptoms attributable to at least one of two diseases during the time that the patient was treated for both infections.
Safety	Adverse drug reactions		Frequency of adverse drug reactions
	Treatment discontinuation due to adverse drug reactions		Patients who stopped treatment because of adverse drug reactions

Comments, editorials, letters to the editor, conference summaries, brief reports, theses, and dissertations will not be considered. Furthermore, studies published in non‐Roman characters will be disregarded.

### 2.3 Data Sources and Search Strategy

A systematic search will be conducted using PubMed, Embase, and Latin American and Caribbean Health Sciences Literature (LILACS) databases. Additionally, a supplementary search of grey literature will be performed using Google Scholar (first 100 records). The search strategies are presented in Table 4. A manual search of the references of included articles may be performed in case further clarification is needed. Search strategies will be modified for each database and include combinations of terms related to histoplasmosis and tuberculosis.

**Table 4:** Search strategies.

PubMed/MEDLINE
#1 “Acquired Immunodeficiency Syndrome”[Mesh] OR “Acquired Immunodeficiency Syndrome*” OR AIDS OR “Acquired Immune Deficiency Syndrome” OR “Acquired Immuno‐Deficiency Syndrome*” OR “Acquired Immuno Deficiency Syndrome” OR “HIV”[Mesh] OR HIV OR HTLV‐III OR “Human Immunodeficiency Virus*” OR “Human T Cell Lymphotropic Virus Type III” OR “Human T‐Cell Lymphotropic Virus Type III” OR “Human T‐Cell Leukemia Virus Type III” OR “Human T Cell Leukemia Virus Type III” OR LAV‐HTLV‐III OR “Lymphadenopathy‐Associated Virus*” OR “Lymphadenopathy Associated Virus” OR “Human T Lymphotropic Virus Type III” OR “Human T‐Lymphotropic Virus Type III” OR “AIDS Virus*” OR “Acquired Immune Deficiency Syndrome Virus” OR “Acquired Immunodeficiency Syndrome Virus”
#2 “Histoplasmosis”[Mesh] OR Histoplasmosis OR Histoplasmoses OR “Histoplasma Infection*” OR “Histoplasma capsulatum Infection*” OR “Disseminated Histoplasmosis” OR “Pulmonary Histoplasmosis” OR “Histoplasma duboisii Infection*” OR “African Histoplasmosis” OR “Cave disease” OR “Darling's disease” OR “*H. capsulatum* infection”
#3 “Mycobacterium tuberculosis”[Mesh] OR “Mycobacterium tuberculosis” OR “Mycobacterium tuberculosis H37Rv” OR “Tuberculosis”[Mesh] OR Tuberculosis OR Tuberculoses OR “Koch's Disease” OR “Koch Disease” OR “Mycobacterium tuberculosis Infection*” OR “Latent Tuberculosis”[Mesh] OR “Latent Tuberculosis” OR “Latent Tuberculosis Infection*” OR “Extensively Drug‐Resistant Tuberculosis”[Mesh] OR “Extensively Drug‐Resistant Tuberculosis” OR “Extensively Drug‐Resistant Tuberculoses” OR XDR‐TB OR “Extremely Drug‐Resistant Tuberculosis” OR “Extremely Drug Resistant Tuberculosis” OR “Tuberculosis, Multidrug‐Resistant”[Mesh] OR “Multidrug‐Resistant Tuberculosis” OR “MDR Tuberculosis” OR “Multi‐Drug Resistant Tuberculosis” OR “Drug‐Resistant Tuberculosis” OR “Bacillus tuberculosis” OR “Bacterium tuberculosis” OR “human tubercle bacillus” OR “Koch bacillus” OR “Koch's bacillus” OR “Mycobacteria tuberculosis” OR “Mycobacterium tuberculosis cultivation” OR “Mycobacterium tuberculosis hominis” OR “Mycobacterium tuberculosis isolation” OR “Mycobacterium tuberculosum” OR “tubercle bacilli” OR “tubercle bacillus” OR “active TB” OR “active tuberculosis” OR “M. tuberculosis infection” OR “minimal tuberculosis” OR “minimum tuberculosis” OR “TB case*” OR “TB disease” OR “TB infection” OR “tuberculous infection” OR “tuberculous lesion” OR “drug resistant TB” OR “resistant pulmonary TB” OR “resistant pulmonary tuberculosis” OR “resistant TB” OR “resistant tuberculosis” OR “extensively drug resistant TB” OR “extensively drug‐resistant (XDR) tuberculosis” OR “extreme drug‐resistant tuberculosis” OR XDRTB OR “latent TB” OR “latent TB infection” OR “latent tuberculosis disease” OR “latent tuberculosis infection” OR “latent tuberculous infection” OR MDR‐TB OR MDR‐tuberculosis OR “multi‐drug resistant pulmonary tuberculosis” OR “multi‐drug resistant TB” OR “multi‐resistant tuberculosis” OR “multidrug resistant pulmonary tuberculosis” OR “multidrug resistant TB” OR “multidrug‐resistant tuberculosis” OR “multiresistant tuberculosis” OR “multidrug resistant tuberculosis” OR “experimental tuberculosis” OR “experimental Mycobacterium tuberculosis infection” OR “experimental tuberculous infection” OR “experimentally induced tuberculosis” OR “experimental tuberculosis” OR “Mycobacterium tuberculosis complex” OR “Mycobacterium tuberculosis group” OR “Mycobacterium tuberculosis complex”
#4 #1 AND #2 AND #3
EMBASE
#1 ‘acquired immune deficiency syndrome’/exp OR ‘acquired human immunodeficiency syndrome’ OR ‘acquired immune deficiency disease syndrome’ OR ‘acquired immuno‐deficiency syndrome’ OR ‘acquired immunodeficiency disease syndrome’ OR ‘acquired immunodeficiency syndrome’ OR ‘acquired immunodeficiency virus syndrome’ OR ‘AIDS’ OR ‘aquired immune deficiency syndrome’ OR ‘aquired immunodeficiency syndrome’ OR ‘HIV/AIDS’ OR ‘human immune deficiency virus/acquired immune deficiency syndrome’ OR ‘human immunodeficiency virus infection/acquired immunodeficiency syndrome’ OR ‘immunodeficiency, acquired’ OR ‘acquired immune deficiency syndrome’ OR ‘Human immunodeficiency virus’/exp OR ‘aids associated lentivirus’ OR ‘aids associated retrovirus’ OR ‘aids associated virus’ OR ‘aids related virus’ OR ‘AIDS virus’ OR ‘HIV’ OR ‘Human immuno deficiency virus’ OR ‘immunodeficiency associated virus’ OR ‘lav’ OR ‘LAV (AIDS)’ OR ‘lymphadenopathy associated retrovirus’ OR ‘Lymphadenopathy associated virus’ OR ‘virus, lymphadenopathy associated’ OR ‘Human immunodeficiency virus’
#2 ‘histoplasmosis’/exp OR ‘Cave disease’ OR ‘Darling disease’ OR ‘Darling's disease’ OR ‘H. capsulatum infection’ OR ‘histo‐plasmosis’ OR ‘Histoplasma capsulatum infection’ OR ‘Histoplasma infection’ OR ‘Histoplasma infections’ OR ‘histoplasmoses’ OR ‘infection by H. capsulatum’ OR ‘infection by Histoplasma’ OR ‘infection by Histoplasma capsulatum’ OR ‘infection caused by Histoplasma capsulatum’ OR ‘infection due to Histoplasma capsulatum’ OR ‘histoplasmosis’
#3 ‘Mycobacterium tuberculosis’/exp OR ‘Bacillus tuberculosis’ OR ‘Bacterium tuberculosis’ OR ‘human tubercle bacillus’ OR ‘Koch bacillus’ OR ‘Koch's bacillus’ OR ‘Mycobacteria tuberculosis’ OR ‘Mycobacterium tuberculosin’ OR ‘Mycobacterium tuberculosis cultivation’ OR ‘Mycobacterium tuberculosis hominis’ OR ‘Mycobacterium tuberculosis isolation’ OR ‘Mycobacterium tuberculosum’ OR ‘tubercle bacilli’ OR ‘tubercle bacillus’ OR ‘tuberculosis hominis, mycobacterium’ OR ‘tuberculosis, mycobacterium’ OR ‘Mycobacterium tuberculosis’ OR ‘tuberculosis’/exp OR ‘active TB’ OR ‘active tuberculosis’ OR ‘case of TB’ OR ‘cases of TB’ OR ‘chronic tuberculosis’ OR ‘infection by M. tuberculosis’ OR ‘infection by Mycobacterium tuberculosis’ OR ‘infection due to M. tuberculosis’ OR ‘infection due to Mycobacterium tuberculosis’ OR ‘infection of M. tuberculosis’ OR ‘infection of Mycobacterium tuberculosis’ OR ‘Koch's disease’ OR ‘M. tuberculosis infection’ OR ‘minimal tuberculosis’ OR ‘minimum tuberculosis’ OR ‘Mycobacterium tuberculosis infection’ OR ‘TB (tuberculosis)’ OR ‘TB case’ OR ‘TB cases’ OR ‘TB disease’ OR ‘TB infection’ OR ‘tuberculous infection’ OR ‘tuberculous lesion’ OR ‘tuberculosis’ OR ‘latent tuberculosis’/exp OR ‘latent TB’ OR ‘latent TB infection’ OR ‘latent tuberculosis disease’ OR ‘latent tuberculosis infection’ OR ‘latent tuberculous disease’ OR ‘latent tuberculous infection’ OR ‘LTBI (tuberculosis)’ OR ‘latent tuberculosis’ OR ‘extensively drug resistant tuberculosis’/exp OR ‘extensively drug resistant TB’ OR ‘extensively drug‐resistant (XDR) tuberculosis’ OR ‘extensively drug‐resistant tuberculosis’ OR ‘extreme drug‐resistant tuberculosis’ OR ‘extremely drug‐resistant tuberculosis’ OR ‘XDR‐TB’ OR ‘XDRTB’ OR ‘extensively drug resistant tuberculosis’ OR ‘multidrug resistant tuberculosis’/exp OR ‘MDR‐TB’ OR ‘MDR‐tuberculosis’ OR ‘MDRTB’ OR ‘multi‐drug resistant (MDR) tuberculosis’ OR ‘multi‐drug resistant pulmonary tuberculosis’ OR ‘multi‐drug resistant TB’ OR ‘multi‐drug resistant tuberculosis’ OR ‘multi‐resistant tuberculosis’ OR ‘multidrug resistant pulmonary tuberculosis’ OR ‘multidrug resistant TB’ OR ‘multidrug‐resistant tuberculosis’ OR ‘multiresistant tuberculosis’ OR ‘tuberculosis, multidrug‐resistant’ OR ‘multidrug resistant tuberculosis’
#4 [embase]/lim NOT ([embase]/lim AND [medline]/lim)
#5 #1 AND #2 AND #3 AND #4
#6 ‘book’/it OR ‘chapter’/it OR ‘conference abstract’/it OR ‘conference paper’/it OR ‘conference review’/it OR ‘data papers’/it OR ‘editorial’/it OR ‘erratum’/it OR ‘letter’/it OR ‘note’/it OR ‘patent’/it OR ‘preprint’/it OR ‘review’/it OR ‘tombstone’/it OR ‘short survey’/it
#7 #5 NOT #6
LILACS
#1 MH: “Acquired Immunodeficiency Syndrome” OR “Acquired Immunodeficiency Syndrome*” OR AIDS OR “Acquired Immune Deficiency Syndrome” OR “Acquired Immuno‐Deficiency Syndrome*” OR “Acquired Immuno Deficiency Syndrome” OR MH:HIV OR HIV OR HTLV‐III OR “Human Immunodeficiency Virus*” OR “Human T Cell Lymphotropic Virus Type III” OR “Human T‐Cell Lymphotropic Virus Type III” OR “Human T‐Cell Leukemia Virus Type III” OR “Human T Cell Leukemia Virus Type III” OR LAV‐HTLV‐III OR “Lymphadenopathy‐Associated Virus*” OR “Lymphadenopathy Associated Virus” OR “Human T Lymphotropic Virus Type III” OR “Human T‐Lymphotropic Virus Type III” OR “AIDS Virus*” OR “Acquired Immune Deficiency Syndrome Virus” OR “Acquired Immunodeficiency Syndrome Virus”
#2 MH: Histoplasmosis OR Histoplasmosis OR Histoplasmoses OR “Histoplasma Infection*” OR “Histoplasma capsulatum Infection*” OR “Disseminated Histoplasmosis” OR “Pulmonary Histoplasmosis” OR “Histoplasma duboisii Infection*” OR “African Histoplasmosis” OR “Cave disease” OR “Darling's disease” OR “H. capsulatum infection”
#3 MH:“Mycobacterium tuberculosis” OR “Mycobacterium tuberculosis” OR “Mycobacterium tuberculosis H37Rv” OR MH:Tuberculosis OR Tuberculosis OR Tuberculoses OR “Koch's Disease” OR “Koch Disease” OR “Mycobacterium tuberculosis Infection*” OR MH:“Latent Tuberculosis” OR “Latent Tuberculosis” OR “Latent Tuberculosis Infection*” OR MH:“Extensively Drug‐Resistant Tuberculosis” OR “Extensively Drug‐Resistant Tuberculosis” OR “Extensively Drug‐Resistant Tuberculoses” OR XDR‐TB OR “Extremely Drug‐Resistant Tuberculosis” OR “Extremely Drug Resistant Tuberculosis” OR MH:“Tuberculosis, Multidrug‐Resistant” OR “Multidrug‐Resistant Tuberculosis” OR “MDR Tuberculosis” OR “Multi‐Drug Resistant Tuberculosis” OR “Drug‐Resistant Tuberculosis” OR “Bacillus tuberculosis” OR “Bacterium tuberculosis” OR “human tubercle bacillus” OR “Koch bacillus” OR “Koch's bacillus” OR “Mycobacteria tuberculosis” OR “Mycobacterium tuberculosis cultivation” OR “Mycobacterium tuberculosis hominis” OR “Mycobacterium tuberculosis isolation” OR “Mycobacterium tuberculosum” OR “tubercle bacilli” OR “tubercle bacillus” OR “active TB” OR “active tuberculosis” OR “M. tuberculosis infection” OR “minimal tuberculosis” OR “minimum tuberculosis” OR “TB case*” OR “TB disease” OR “TB infection” OR “tuberculous infection” OR “tuberculous lesion” OR “drug resistant TB” OR “resistant pulmonary TB” OR “resistant pulmonary tuberculosis” OR “resistant TB” OR “resistant tuberculosis” OR “extensively drug resistant TB” OR “extensively drug‐resistant (XDR) tuberculosis” OR “extreme drug‐resistant tuberculosis” OR XDRTB OR “latent TB” OR “latent TB infection” OR “latent tuberculosis disease” OR “latent tuberculosis infection” OR “latent tuberculous infection” OR MDR‐TB OR MDR‐tuberculosis OR “multi‐drug resistant pulmonary tuberculosis” OR “multi‐drug resistant TB” OR “multi‐resistant tuberculosis” OR “multidrug resistant pulmonary tuberculosis” OR “multidrug resistant TB” OR “multidrug‐resistant tuberculosis” OR “multiresistant tuberculosis” OR “multidrug resistant tuberculosis” OR “experimental tuberculosis” OR “experimental Mycobacterium tuberculosis infection” OR “experimental tuberculous infection” OR “experimentally induced tuberculosis” OR “experimental tuberculosis” OR “Mycobacterium tuberculosis complex” OR “Mycobacterium tuberculosis group” OR “Mycobacterium tuberculosis complex”
#4 #1 AND #2 AND #3
Google Scholar
(HIV OR AIDS) AND (“Mycobacterium tuberculosis” OR Tuberculosis) AND Histoplasmosis

### 2.4 Study Selection and Data Extraction

Studies retrieved from selected databases will be allocated into Rayyan for duplicate removal, as well as for screening and selection.

Two independent researchers will carry out an initial screening stage by independently examining the title and abstract of all records retrieved, to identify potentially relevant articles. Subsequently, a second selection stage will be carried out by two independent researchers, who will review the full‐text articles to determine their eligibility to the predefined criteria.

Data extraction will be performed by two independent reviewers, and capture information regarding: the studies (authors, year of publication, country of conduct, study design, study setting, timeframe for follow‐up and sources of funding); the population studied (sociodemographic and clinical characteristics); the interventions used (medication and dosage regimen); comparators (medication and dosage regimen); the outcomes evaluated (clinical improvement, mortality, relapse, adverse drug reactions, and treatment discontinuation due to adverse drug reactions) and their respective results.

Disagreements during the study selection and data extraction stages will be evaluated by a third researcher and resolved through consensus. Additionally, a list of potentially relevant studies that were read in full‐text form but excluded from the review will be provided in the final report, along with exclusion justification.

### 2.5 Risk of Bias and Strength of Recommendation

The risk of bias will be assessed through different tools depending on the study design. For randomized controlled trials, the risk of bias will be assessed using the Cochrane RoB 2 tool [12]. The risk of bias for non‐randomized interventional studies will be assessed using the ROBINS‐I tool [13]. The risk of bias for cohort and case‐control studies will be assessed using the Newcastle Ottawa Scale (NOS) [14]. Lastly, other study designs such as cross‐sectional studies, case series, or case reports will be assessed using appropriate Joanna Briggs Institute (JBI) critical appraisal tools [15].

The strength of the body of evidence will be evaluated using the Grading of Recommendations Assessment, Development and Evaluation (GRADE) system for each outcome [16].

Both the risk of bias and the quality of evidence assessments will be performed by two independent reviewers. Any disagreements will be evaluated by a third researcher and resolved through consensus.

### 2.6 Statistical Analysis

Meta‐analyses will be performed using the Comprehensive Meta‐Analysis v.2 software (Biostat, Englewood, NJ). Single‐arm meta‐analyses will be used to report individual intervention outcomes as event rate (%) with a 95% confidence interval (CI). In direct comparative meta‐analyses (pairwise), dichotomous and continuous results will be described as relative risk (RR) or odds ratio (OR) and mean difference (MD), respectively, also with 95% CI. Anticipating the existence of heterogeneity between studies, the random‐effects model will be employed. The inverse variance and Mantel–Haenszel statistical methods will be applied to continuous and dichotomous data, respectively.

Heterogeneity between studies will be assessed using the Higgins inconsistency test (*I* ^2^) and will be considered significant when *p* < 0.05 and high when *I* ^2^ > 75% [17]. Subgroup analysis will be conducted to identify studies responsible for heterogeneity, considering study design, sociodemographic and clinical characteristics of the population, interventions, and dosage regimens.

If a meta‐analysis is not feasible, a structured narrative synthesis of the findings will be conducted. Reasons for not performing a meta‐analysis may include substantial heterogeneity in study design, interventions, or outcome measures, or insufficient data for quantitative synthesis.

## 3 Conclusion

This systematic review will provide a detailed analysis of the efficacy, effectiveness, and safety of different regimens for treating patients with HIV/AIDS coinfected with histoplasmosis‐TB, helping identify the most effective and safe therapeutic options for managing these infectious conditions. By addressing an important gap in the scientific literature, therefore, the findings of this review may contribute to the improvement of clinical practice for the drug treatment of endemic and neglected clinical conditions. Ultimately, this can result in better clinical outcomes, such as greater clinical improvement, as well as fewer adverse events. Thus, the findings of this review may positively impact public health, stimulating the implementation of evidence‐based practices and guiding the adoption of specific treatment protocols for coinfected patients in endemic regions.

## Author Contributions

**Beatriz Bernava Sarinho:** writing – original draft, writing – review and editing. **Inajara Rotta:** conceptualization, methodology, writing – review and editing. **Tácio de Mendonça Lima:** conceptualization, methodology, writing – review and editing. **Brígida Dias Fernandes:** conceptualization, methodology, writing – review and editing. **Patricia Melo Aguiar:** conceptualization, methodology, writing – review and editing. **Marília Berlofa Visacri:** conceptualization, methodology, project administration, supervision, writing – review and editing.

## Funding

The authors received no specific funding for this work.

## Ethics Statement

The authors have nothing to report.

## Conflicts of Interest

The authors declare no conflicts of interest.

## Transparency Statement

The corresponding author, Marília Berlofa Visacri, affirms that this manuscript is an honest, accurate, and transparent account of the study being reported; that no important aspects of the study have been omitted; and that any discrepancies from the study as planned (and, if relevant, registered) have been explained.

## Data Availability

The authors have nothing to report.

## References

[1] M. P. N. Kuate , B. E. Ekeng , R. Kwizera , C. Mandengue , and F. Bongomin , “Histoplasmosis Overlapping With HIV and Tuberculosis in Sub‐Saharan Africa: Challenges and Research Priorities,” Therapeutic Advances in Infectious Disease 8, no. 20499361211008675 (2021): 1–7, 10.1177/20499361211008675. PMC8040546 33889408

[2] G. Aguilar , G. Lopez , O. Sued , et al., “Implementation of a Rapid Diagnostic Assay Package for Cryptococcosis, Histoplasmosis and Tuberculosis in People Living With HIV in Paraguay,” BMC Infectious Diseases 24, no. 406 (2024): 406, 10.1186/s12879-024-09257-5. 38627642 PMC11020460

[3] S. L. S. Souza , P. V. S. Feitoza , J. R. Araújo , R. V. Andrade , and L. C. L. Ferreira , “Causas De Óbito Em Pacientes Com Síndrome Da Imunodeficiência Adquirida, Necropsiados Na Fundação De Medicina Tropical Do Amazonas,” Revista da Sociedade Brasileira de Medicina Tropical 41, no. 3 (2008): 247–251, 10.1590/S0037-86822008000300005. 18719803

[4] C. Murillo , A. M. Tobón , A. Restrepo , et al., “Tuberculosis and Histoplasmosis Co‐Infection in AIDS Patients,” American Journal of Tropical Medicine and Hygiene 87, no. 6 (2012): 1094–1098, 10.4269/ajtmh.2012.12-0292. 23128292 PMC3516081

[5] “Consolidated Guidelines on HIV Prevention, Testing, Treatment, Service Delivery and Monitoring: Recommendations for a Public Health Approach,” World Health Organization (WHO), accessed July 17, 2025, https://www.who.int/publications/i/item/9789240031593. 34370423

[6] “WHO Consolidated Guidelines on Tuberculosis. Module 4: Treatment and Care,” World Health Organization (WHO), accessed July 17, 2025, https://www.who.int/publications/i/item/9789240107243. 40163610

[7] “Guidelines for Diagnosing and Managing Disseminated Histoplasmosis Among People Living With HIV [Procedures, manuals, guidelines],” PAHO, published 2020, https://iris.paho.org/handle/10665.2/52304. 36475570

[8] “Guidelines for the Use of Antiretroviral Agents in Adults and Adolescents With HIV,” Department of Health and Human Services (HHS), accessed February 23, 2025, https://clinicalinfo.hiv.gov/sites/default/files/guidelines/documents/adult-adolescent-arv/guidelines-adult-adolescent-arv.pdf.

[9] M. J. Page , J. E. McKenzie , P. M. Bossuyt , et al., “The PRISMA 2020 Statement: An Updated Guideline for Reporting Systematic Reviews,” BMJ 372, no. 71 (2021): n71, 10.1136/bmj.n71. 33782057 PMC8005924

[10] B. Acharya , A. Acharya , S. Gautam , et al., “Advances in Diagnosis of Tuberculosis: An Update Into Molecular Diagnosis of *Mycobacterium Tuberculosis* ,” Molecular Biology Reports 47 (2020): 4065–4075, 10.1007/s11033-020-05413-7. 32248381

[11] M. A. Toscanini , A. D. Nusblat , and M. L. Cuestas , “Diagnosis of Histoplasmosis: Current Status and Perspectives,” Applied Microbiology and Biotechnology 105 (2021): 1837–1859, 10.1007/s00253-021-11170-9. 33587157

[12] J. A. C. Sterne , J. Savović , M. J. Page , et al., “RoB 2: A Revised Tool for Assessing Risk of Bias in Randomised Trials,” BMJ 366, no. i4898 (2019): l4898, 10.1136/bmj.l4898. 31462531

[13] J. A. Sterne , M. A. Hernán , B. C. Reeves , et al., “ROBINS‐I: A Tool for Assessing Risk of Bias in Non‐Randomised Studies of Interventions,” BMJ 355, no. i4919 (2016): i4919, 10.1136/bmj.i4919. 27733354 PMC5062054

[14] G. A. Wells , B. Shea , D. O'Connell , et al., “The Newcastle‐Ottawa Scale (NOS) for Assessing the Quality of Nonrandomized Studies in Meta‐Analyses, accessed February 23, 2025, http://www.ohri.ca/programs/clinical_epidemiology/oxford.asp.

[15] Joanna Briggs Institute (JBI), “JBI Critical Appraisal Tools,” JBI Global, accessed December 12, 2025, https://jbi.global/critical-appraisal-tools.

[16] G. Guyatt , A. D. Oxman , E. A. Akl , et al., “GRADE Guidelines: 1. Introduction‐Grade Evidence Profiles and Summary of Findings Tables,” Journal of Clinical Epidemiology 64, no. 4 (2011): 383–394, 10.1016/j.jclinepi.2010.04.026. 21195583

[17] J. P. T. Higgins , J. Thomas , J. Chandler , et al., eds., “Cochrane Handbook for Systematic Reviews of Interventions, version 6.5,” accessed February 23, 2025, www.training.cochrane.org/handbook.

